# Active but not passive passengers: Persistent-propagative plant viruses manipulate insect vectors for efficient transmission

**DOI:** 10.1371/journal.ppat.1014368

**Published:** 2026-06-26

**Authors:** De-Shui Liu, Dong-min Gao, Xian-Bing Wang, Qiang Gao

**Affiliations:** 1 Beijing Life Science Academy, Beijing, China; 2 State Key Laboratory of Plant Environmental Resilience, College of Biological Sciences, China Agricultural University, Beijing, China; 3 College of Grassland Science and Technology, China Agricultural University, Beijing, China; The Ohio State University, UNITED STATES OF AMERICA

## Abstract

Most plant viruses rely on insect vectors for transmission. In general, nonviruliferous vectors acquire viruses from infected plants, whereas viruliferous vectors subsequently move to uninfected plants for virus inoculation. However, they are not merely passive passengers within their vector. Persistent-propagative plant viruses, which invade and replicate in insect vectors, can actively and directly reshape their physiology, morphology, and feeding behavior to facilitate efficient transmission. Recent studies have uncovered both indirect plant-mediated mechanisms and direct vector-targeted mechanisms, including modulation of neural signaling, olfactory systems, wing development, and feeding activity. Here, we summarize current understanding of how persistent-propagative plant viruses integrate indirect plant-mediated effects with direct manipulation of insect vectors. We further discuss the ecological implications of these virus–plant–vector interactions, highlight major bottlenecks in virus–insect interaction research, and emerging technological advances that may facilitate future mechanistic studies and support innovative strategies for controlling vector-borne plant viral diseases.

## Introduction

As obligate intracellular parasites, most animal and plant viruses lack autonomous motility and usually rely on various insect vectors for transmission. For instance, dengue virus (DENV) and Zika virus (ZIKV) induce N-acetyl-L-tyrosine accumulation in mosquito heads, activating the tyrosine–dopamine pathway and thereby elevating dopamine levels to enhance locomotion and blood-feeding propensity. They also disrupt the circadian rhythm of *Aath*, which encodes tyrosine hydroxylase, sustaining dopamine biosynthesis [1]. Similar to animal viruses, more than 70% of plant viruses are transmitted by various insect vectors [2]. Through long-term coevolution, plant viruses have selected the most efficient vector species, a process that appears almost active and purposeful [3]. Most plant viruses have evolved to exploit the feeding behavior and morphology of their insect vectors as an efficient transmission strategy, effectively “hitchhiking” insect vectors to enhance dispersal and increase epidemic potential [4]. Plant viruses are transmitted by insect vectors via nonpersistent, semi-persistent, and persistent modes, determined by the duration of viral particles within the vectors. Nonpersistent viruses such as cucumber mosaic virus (CMV) and semi-persistent viruses such as cauliflower mosaic virus (CaMV) are transmitted in a noncirculative manner, during which virions bind to specific cuticular structures within the insect stylet or foregut [5–7]. These viruses are acquired and transmitted within a short period. In contrast, persistent transmission occurs in a circulative manner, during which the viruses cross multiple biological barriers by entering the epithelial cells of the insect midgut, traversing the hemolymph, and ultimately reaching the salivary glands [2,7]. Persistent viruses are further classified into two types, propagative viruses which replicate within insect tissues, such as the rhabdovirus barley yellow striate mosaic virus (BYSMV) and the tenuivirus rice stripe virus (RSV) [8–10]; and nonpropagative viruses, which circulate through the vector body without replication, such as the luteovirus barley yellow dwarf virus (BYDV) [4,6,11].

For successful transmission and epidemic spread, plant viruses must not only trigger attraction of appropriate insect vectors to infected plants to maximize viral acquisition, but also enhance vector settling and feeding, as well as subsequently facilitate efficient release and inoculation into new host plants. During these processes, viruses actively manipulate both plant hosts and insect vectors to facilitate their transmission. Increasing evidence indicates that plant viruses indirectly influence vector behavior by reprogramming host phytohormone signaling and secondary metabolism, which can increase vector attraction, improve host suitability, and promote virus acquisition. For instance, in plants infected with *Begomovirus*, the βC1 protein encoded by the associated β-satellite DNA interacts with the plant transcription factor MYC2 to suppress the expression of terpene synthase genes, thereby reducing plant resistance to the whitefly vector [12]. βC1 also interacts with the vascular-specific transcription factor WRKY20, disrupting its dimerization and suppressing its transcriptional activity. This interaction leads to increased accumulation of aliphatic glucosinolates in nonvascular leaf tissues, accompanied by a reduction in indole glucosinolates within leaf veins [13]. Such metabolic reprogramming inhibits nonvector insects but benefits vector whiteflies, thereby facilitating virus transmission [13]. Similarly, tomato chlorosis virus (ToCV) infection promotes whitefly attraction and feeding by altering host physiology. The viral P9 protein interacts with light-harvesting complex I subunit Lhca4 (Lhca4), causing chlorophyl degradation and increased accumulation of the volatile neophytadiene for whitefly attractiveness. In addition, ToCV suppresses jasmonic acid (JA) signaling, further facilitating vector feeding and virus spread [14]. These mechanisms have been extensively summarized in recent reviews, which demonstrate that viruses indirectly modulate their insect vectors through the manipulation of plant hosts [15–21].

Although it has long been recognized that plant viruses can directly manipulate their insect vectors, the molecular mechanisms underlying these interactions have remained largely obscure [17]. Persistent-propagative plant viruses include negative-sense RNA viruses, such as rhabdoviruses, orthotospoviruses, and tenuiviruses, double-stranded RNA viruses in the family *Reoviridae* [22], and geminiviruses such as TYLCV, whose replication in insect vectors was previously debated but has recently been supported by experimental evidence [23,24]. Unlike nonpropagative viruses, these viruses can replicate within insect vectors and synthesize viral RNAs and proteins, as they do in plant cells, thereby establishing more active and intimate interactions with their vectors [25]. Recent studies have revealed that several of these viruses can directly modulate vector behavior by altering wing morphology, neural signaling, olfactory perception, and feeding activity [26–28]. In this review, we summarize recent advances in understanding how persistent-propagative plant viruses manipulate insect vectors through both indirect plant-mediated and direct vector-targeted mechanisms, and discuss the implications for plant virus–vector interactions and disease epidemiology.

## Plant rhabdoviruses coordinate neurotropic infection–mediated circadian regulation and jasmonate signaling to manipulate vector behavior

Plant rhabdoviruses, members of the subfamily *Betarhabdovirinae* within the family *Rhabdoviridae*, currently comprise 253 classified species [29,30], and possess negative-sense RNA genomes that may be nonsegmented, bipartite, or tripartite [31]. Most plant rhabdoviruses replicate in their arthropod vectors and are transmitted in a persistent-propagative manner [32]. Increasing evidence indicates that these viruses establish neurotropic infections in their insect hosts. Early studies showed that maize mosaic virus (MMV) and maize fine streak virus (MFSV) systemically infect the nervous systems of their insect vectors, suggesting that a neurotropic route may help these viruses overcome transmission barriers [33,34]. Subsequent work further demonstrated that the matrix protein of rice yellow stunt virus (RYSV) interacts with axonal microtubules and facilitates the transport of nonenveloped viral structures through the central nervous system, thereby promoting viral dissemination to the salivary glands and efficient transmission [35].

Animal rhabdoviruses such as rabies virus usually cause significant cytopathological damage to host nervous systems [36]. By contrast, plant rhabdoviruses appear to have evolved a more balanced relationship with their insect vectors. Recent research has revealed that the Hikaru genki homolog (NcHig), a neural factor in leafhoppers, binds to the M proteins of both RYSV and rice stripe mosaic virus (RSMV), suppressing viral infection in neural tissues [37]. This interaction likely represents a co-evolutionary adaptation, whereby a stealth strategy maintains vector fitness to promote viral persistence. Thus, neurotropism suggests that plant rhabdoviruses can disseminate through the insect nervous system to reach the salivary glands, providing an alternative to the classical midgut–hemolymph–salivary gland route and resembling neuroinvasive routes used by some animal rhabdoviruses [38].

**Fig 1:**
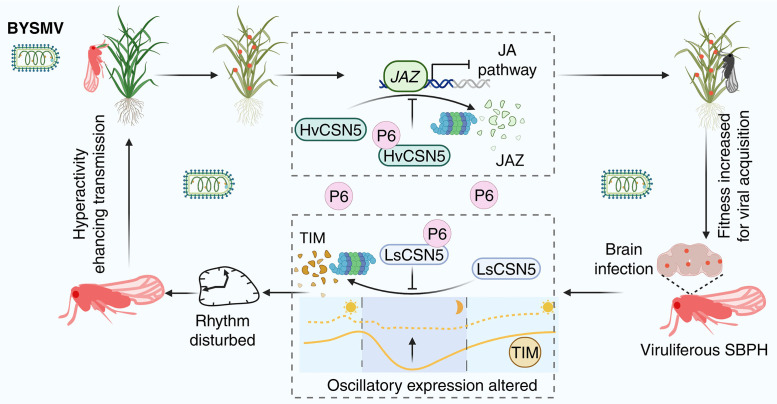
BYSMV manipulates insect vector behavior through indirect and direct mechanisms. In BYSMV-infected barley plants, the viral P6 protein interacts with HvCSN5, a subunit of the COP9 signalosome, thereby stabilizing the jasmonate (JA) signaling repressor JAZ and suppressing JA-mediated anti-insect defense. This plant-mediated effect facilitates feeding by small brown planthoppers (SBPHs) and promotes virus acquisition (indirect manipulation). In viruliferous insects, BYSMV replicates in the central nervous system, where P6 binds to LsCSN5, the insect homolog of HvCSN5, disrupts the rhythmic expression of the circadian clock protein TIM, and enhances vector locomotor activity and feeding behavior (direct manipulation). Together, these processes form a dual-host manipulation strategy that maximizes BYSMV transmission. This figure was created in BioRender, https://BioRender.com/gydn1yh.

In addition to facilitating systemic spread within insect vectors, viral infection of the nervous system can also influence vector circadian rhythm and feeding behavior. BYSMV, an economically important cytorhabdovirus, is dependent on the small brown planthopper (SBPH) for transmission and infects more than 26 crop species in the field [39]. The accessory protein P6 of BYSMV interacts with the barley COP9 signalosome subunit 5 (HvCSN5), and thereby suppresses LsCSN5-regulated de-neddylation of Cullin 1 (CUL1). Consequently, inhibition of CUL1-based E3 ligase-mediated degradation of JAZ proteins suppresses jasmonate signaling, thereby enhancing vector attraction and virus acquisition [40]. Consistent with other animal and plant rhabdoviruses, BYSMV has been shown to infect the brains of its insect vectors [8]. Interestingly, BYSMV P6 also interacts with the insect COP9 signalosome subunit 5 (LsCSN5), a homolog of HvCSN5, and suppresses LsCSN5-regulated de-neddylation of Cullin 1 (CUL1). This inhibition prevents CUL1-based E3 ligase-mediated degradation of the circadian clock protein Timeless (TIM), thereby disrupting circadian rhythm and enhancing locomotor activity in infected insects, which increases the probability of viral transmission [28]. Thus, BYSMV uses the same viral effector to target homologous CSN5–CUL1 ubiquitin-system components in both plants and insects, indirectly promoting vector attraction through plant jasmonate signaling and directly enhancing vector transmission activity through disruption of the insect circadian clock (Fig 1).

Similarly, RSMV also exploits the host ubiquitin-system to create favorable ecological conditions for viral persistence and spread. The RSMV P6 protein interacts with the rice heading-related E3 ubiquitin ligase Heading date Associated Factor 1 (HAF1), impairing its function and delaying rice heading. This virus-induced developmental change creates a more favorable environment for leafhopper vectors and viruses to overwinter [41], thereby facilitating long-term virus maintenance and subsequent transmission.

## Plant bunyaviruses manipulate stylet permeability, hormone signaling, and wing development to enhance vector-mediated transmission

Beyond plant rhabdoviruses, several other persistent-propagative plant negative-sense RNA viruses are also major threats to crop production, including rice RSV and rice grassy stunt virus (RGSV) in the genus *Tenuivirus*, and tomato spotted wilt virus (TSWV) in the genus *Orthotospovirus*, all of which belong to the class *Bunyaviricetes* [42]. Recent studies, particularly those on RSV, have begun to reveal how these viruses manipulate vector behavior and transmission through both indirect plant-mediated and direct vector-targeted mechanisms.

**Fig 2:**
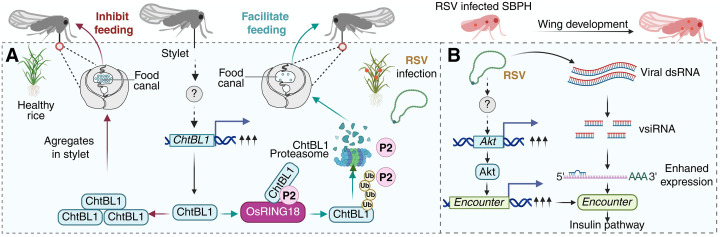
Plant bunyavirus RSV manipulates stylet permeability and wing development to enhance vector-mediated transmission. **(A)** During feeding, SBPHs induce expression of ChtBL1, which enters the stylet food canal, binds chitin, and forms inclusion bodies that restrict stylet permeability and suppress feeding. Upon RSV infection, the viral P2 protein mediates E3 ubiquitin ligase-dependent degradation of ChtBL1, increasing stylet permeability and facilitating viral transmission (indirect manipulation). **(B)** RSV replication in SBPHs upregulates the insulin pathway kinase Akt, which in turn induces expression of Encounter, a planthopper-specific protein. An RSV-derived siRNA also acts as a microRNA targeting the 5′untranslated region of Encounter, enhancing its transcription. These two synergistic effects elevate Encounter expression and drive the development of long-winged male morphs that favor viral spread (direct manipulation). This figure was created in BioRender, https://BioRender.com/1txyyud.

The stylet of insect vectors serves as both the entry and exit conduit for plant viruses during acquisition and inoculation. It consists of a food canal and a salivary canal, which merge at the tip into a common duct. This architecture is mainly composed of cuticular chitin [43]. Recent studies have revealed that plant chitin-binding lectins (ChtBLs) function as defensive factors against insect feeding. In rice, SBPH feeding strongly induces *OsChtBL1*, which encodes a protein with four tandem chitin-binding domains. OsChtBL1 binds to stylet chitin and forms large aggregates, producing dense mesh-like deposits within the food canal that act as a physical barrier to virus acquisition and inoculation. To overcome the feeding barrier, RSV has evolved a specific suppression mechanism through inhibiting the accumulation of plant OsChtBL1. Mechanistically, the RSV P2 protein links OsChtBL1 to the host RING-type E3 ubiquitin ligase OsRING18, thereby promoting the ubiquitination and subsequent proteasome-dependent degradation of OsChtBL1. This degradation decreases OsChtBL1 deposition within the stylet food canal, ultimately facilitating viral acquisition and transmission [44] (Fig 2A). These findings reveal a plant-mediated strategy by which RSV remodels the vector feeding interface, suggesting that the insect stylet is not only a passive conduit for virus movement but also a critical target in virus–plant–vector interactions.

RSV can also directly manipulate vector morphology to facilitate transmission. Insect wings exhibit typical phenotypic plasticity under different environmental conditions. For instance, brown planthoppers develop either short-winged or long-winged morphs depending on host plant quality, a process controlled by insulin receptor-mediated nutrient-sensing pathways [45]. Virus infection can also alter wing morph determination in insect vectors to facilitate long-distance transmission. RSV specifically induces the development of long-winged morphs in male insects. Mechanistically, an RSV-derived small interfering RNA (vsiRNA) targets the 5′ untranslated region (UTR) of the *Encounter* gene and improves its expression. *Encounter* functions as a downstream effector of Akt within the insulin signaling pathway. RSV-induced upregulation of *Akt* enhances *Encounter* expression and promotes the development of the long-winged phenotype [26] (Fig 2B).

Modulation of wing development has also been observed in the nonpersistent virus CMV. CMV infection is often associated with satellite RNAs (satRNAs) [46], among which Y-satellite RNA (Y-sat) induces leaf yellowing by producing a vsiRNA that targets magnesium protoporphyrin chelatase subunit I (*ChlI*), a key enzyme in chlorophyl biosynthesis. Degradation of *ChlI* mRNA reduces chlorophyl accumulation and enhances the visual attraction of aphid vectors [47,48]. During feeding, Y-sat double-stranded RNA can enter aphids and be processed into siRNAs. One Y-sat-derived 24-nt siRNA mimics aphid miR-9b and competitively binds the mRNA of the wing development-related gene *ABCG4*, relieving miR-9b-mediated repression and upregulating *ABCG4* expression to promote wing formation [48]. This dual “pull–push” strategy enables CMV/Y-sat to attract aphid vectors and promote their dispersal, thereby enhancing transmission efficiency and epidemic spread. Unlike RSV-derived vsiRNAs, which are produced during viral replication within insect vectors, CMV/Y-sat-derived siRNAs originate from plant-derived dsRNAs that are ingested and processed by aphids. Nevertheless, both mechanisms ultimately regulate vector wing development to facilitate virus transmission.

Plant bunyaviruses also target hormone signaling to manipulate vector behavior and transmission ecology. RSV NS2 interacts with OsMYC2 and sequesters it in the cytoplasm, thereby blocking OsMYC2-mediated expression of *OsBSMT1* and methyl salicylate (MeSA) biosynthesis, thereby impairing the recruitment of parasitoids that attack insect vectors. By reducing natural enemy attraction, RSV promotes vector persistence on infected plants and indirectly supports viral transmission from a broader ecological niche perspective [49]. MYC transcription factors are also targeted by TSWV. TSWV NSs protein directly interacts with MYC2 and its close homologs MYC3 and MYC4, thereby suppressing JA-mediated activation of terpene synthase genes. This reduces the biosynthesis of repellent volatile monoterpenes, increasing plant attractiveness to thrips vectors and promoting virus spread [50]. Together, these studies suggest that plant viruses manipulate insect vector behavior through complex, multilayered regulatory networks rather than a single linear pathway, thereby reshaping both vector preference and the broader ecological context of transmission.

## Plant reoviruses reshape vector behavior by reprogramming plant volatiles and modulating vector olfaction

Several major rice viral diseases are caused by plant reoviruses, including rice dwarf virus (RDV), rice gall dwarf virus (RGDV), rice black-streaked dwarf virus (RBSDV), and southern rice black-streaked dwarf virus (SRBSDV). These viruses possess 10–12 segmented double-stranded RNA genomes and are transmitted by leafhopper or planthopper vectors in a persistent-propagative manner [51]. RDV, a member of the genus *Phytoreovirus*, is transmitted by green rice leafhoppers (GRLHs, *Nephotettix cincticeps*) and manipulates vector behavior by altering rice volatile emissions. RDV-infected plants emit increased levels of specific volatiles, including E-β-caryophyllene and 2-heptanol. E-β-caryophyllene attracts nonviruliferous GRLHs to settle on infected plants, thereby facilitating virus acquisition, but has little effect on viruliferous GRLHs. In contrast, 2-heptanol repels viruliferous GRLHs from infected plants while having little effect on nonviruliferous insects [52]. Similarly, SRBSDV, a member of the genus *Fijivirus*, is transmitted by the white-backed planthopper (WBPH, *Sogatella furcifera*). Nonviruliferous WBPHs preferentially settle on SRBSDV-infected rice plants to acquire the virus, whereas viruliferous individuals shift their preference toward healthy plants, thereby promoting viral inoculation and spread [53,54].

**Fig 3:**
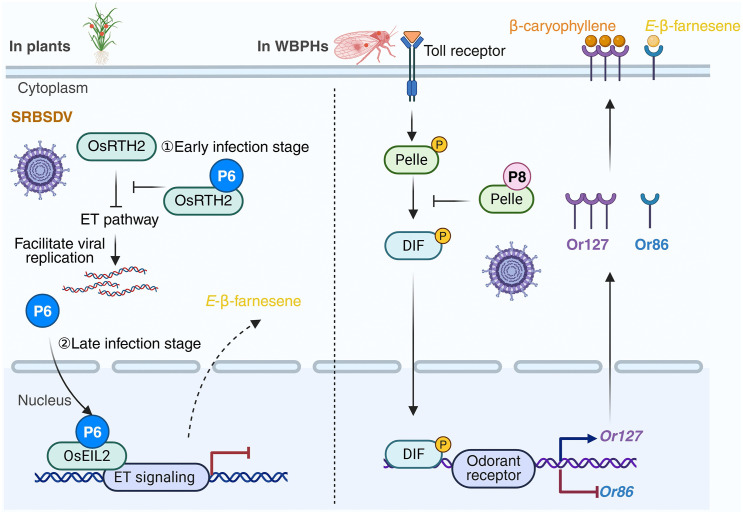
Plant reovirus SRBSDV reshapes vector behavior by reprogramming plant volatiles and modulating vector olfaction. In SRBSDV-infected rice plants, the viral P6 protein exhibits stage-specific localization and function. During early infection, P6 localizes to the cytoplasm, where it interacts with the ET negative regulator OsRTH2, thereby activating the ET signaling pathway and promoting viral replication. At later stages, P6 translocates into the nucleus and binds to the ET positive regulator OsEIL2, suppressing ET signaling, which may enhance the production of the volatile E-β-farnesene that may be favored by healthy WBPHs (indirect manipulation). In SRBSDV-infected vectors, the viral P8 protein interacts with Pelle, a key kinase in the Toll pathway, inhibiting the phosphorylation and nuclear translocation of the transcription factor DIF. DIF suppression perturbs odorant receptor gene regulation, leading to the downregulation of Or86 (responsive to E-β-farnesene) and the upregulation of Or127 (β-caryophyllene). Consequently, viruliferous insects display altered olfactory sensitivity, enabling them to distinguish between infected and healthy rice plants and thereby promoting efficient virus transmission (direct manipulation). This figure was created in BioRender, https://BioRender.com/mh8up2p.

In plants, during early infection, the SRBSDV P6 protein localizes to the cytoplasm, where it interacts with ethylene-response repressor OsRTH2 to enhance ethylene signaling and promote SRBSDV replication. At the later stage, P6 translocates into the nucleus and interacts with the key ethylene signaling component OsEIL2, thereby suppressing ethylene signaling and enhancing the attractiveness of infected plants to vectors [55]. Olfactory perception plays a central role in shaping insect development and behavior [56]. In insects, SRBSDV infection shifts vector preference from infected to healthy rice plants for efficient virus transmission. Mechanistically, the viral P8 protein competitively binds to the kinase Pelle, suppressing Toll signaling and consequently increasing viral infection. Moreover, this inhibition prevents phosphorylation and nuclear translocation of DIF, an important transcription factor of Toll signaling, leading to downregulation of the olfactory receptor *Or86* and upregulation of the receptor *Or127* in the insect olfactory system. Consequently, nonviruliferous planthoppers exhibit enhanced antennal responses to E-β-farnesene by Or86, whereas viruliferous individuals were more sensitive to β-caryophyllene by Or127. By perturbing the Toll-DIF regulatory pathway, SRBSDV reprograms olfactory receptor gene expression and reshapes vector sensory perception, thereby driving a behavioral shift that favors viral acquisition and dissemination [27,57] (Fig 3). Future work should investigate how ethylene signaling interacts with volatile biosynthesis pathways and how the two volatiles E-β-farnesene and β-caryophyllene differ between healthy and virus-infected plants to clarify their roles in mediating vector behavior.

Although SRBSDV-induced changes in rice volatile profiles have not yet been directly demonstrated, RDV studies show that viral infection increases (E)-β-caryophyllene emission, attracting nonviruliferous GRLHs but not viruliferous ones. By contrast, SRBSDV-infected WBPHs become more sensitive to β-caryophyllene through Or127 upregulation, suggesting that β-caryophyllene-related cues are differentially perceived across vector species and infection statuses.

## Geminiviruses coordinate plant volatile cues, insect olfactory receptors, and neural signaling to reshape vector behavior

**Fig 4:**
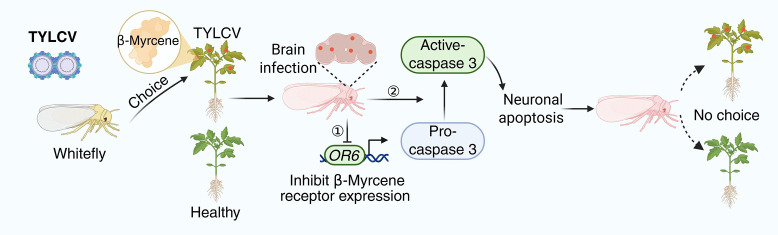
Geminivirus TYLCV reshapes vector behavior through plant volatile cues, olfactory receptor modulation, and neural signaling. TYLCV-infected tomato plants emit higher levels of the volatile β-myrcene, which increases their attractiveness to the primary vector, the whitefly *Bemisia tabaci* MED (indirect manipulation). In viruliferous whiteflies, TYLCV suppresses the expression of the β-myrcene receptor BtOR6 while simultaneously activating caspase-dependent neuronal apoptosis. Together, these processes abolish host preference in viruliferous insects, driving their dispersal from infected to healthy plants and thereby facilitating TYLCV transmission (direct manipulation). This figure was created in BioRender. https://BioRender.com/gydn1yh.

Geminiviruses are economically important plant single-stranded DNA viruses that are traditionally considered to be transmitted by whiteflies, leafhoppers, or aphids in a circulative-persistent manner [58–60]. However, recent evidence that TYLCV, a representative member of the genus *Begomovirus*, can replicate within whiteflies suggests that its transmission may also exhibit propagative features [23]. TYLCV-free *Bemisia tabaci* MED (Mediterranean genetic group) preferentially settle on TYLCV-infected tomato plants rather than healthy plants, likely attributable to virus infection-mediated modifications of plant volatiles [61]. By contrast, TYLCV-infected whiteflies lose this preference and no longer discriminate between infected and healthy tomatoes, indicating that the virus directly modulates vector feeding behavior. TYLCV employs a dual-level manipulation strategy that integrates host volatile modulation with vector sensory reprogramming to enhance transmission. TYLCV-infected plants significantly increase the emission of the monoterpene β-myrcene, which enhances their attractiveness to virus-free *Bemisia tabaci* MED through the odorant receptor BtMEDOR6. Silencing *BtMEDOR6* abolishes the innate preference of naïve MED for infected plants, confirming its essential role in volatile detection. Intriguingly, after virus acquisition, *BtMEDOR6* expression is markedly downregulated, resulting in loss of the preference for different host plants to facilitate insect vector dispersal from infected plants to healthy hosts [62] (Fig 4).

As a direct vector-targeted mechanism, TYLCV infection also triggers apoptotic neurodegeneration in the whitefly brain, characterized by vacuolar neuropathological lesions reminiscent of those described in Alzheimer’s and Parkinson’s disease models [63]. This neurodegeneration neutralizes host preference after virus acquisition, thereby biasing viruliferous vectors toward healthy plants and completing the transmission cycle. Mechanistically, TYLCV infection induces Caspase 3b cleavage into its active homodimer, resulting in apoptotic neurodegeneration, whereas silencing Caspase 1 or Caspase 3b mitigates TYLCV-induced loss of host preference in free-choice assays [62] (Fig 4). Collectively, TYLCV first attracts naïve vectors to infected plants through altered plant volatiles and then neutralizes host preference after acquisition by altering olfactory receptor expression and inducing neurodegeneration, thereby optimizing viruliferous vector behavior for efficient transmission. Whether replication within insect vectors also occurs in other geminiviruses remains unresolved, but this question may shift the field from viewing geminiviruses as circulative-persistent viruses toward recognizing potential persistent-propagative features.

## Knowledge gaps and future perspectives

Accumulating evidence supports the conclusion that persistent-propagative plant viruses reshape their vectors at multiple levels, from morphology to behavior, to optimize transmission. Over the past decade, the molecular mechanisms underlying these phenomena have begun to be elucidated. Despite these advances, several challenges remain in experimental systems, technological platforms, and field-relevant ecological complexity.

## Expanding reverse genetics systems for plant NSR and dsRNA viruses

Most plant negative-strand RNA (NSR) viruses and double-stranded RNA (dsRNA) viruses are transmitted by insect vectors in a persistent-propagative manner. However, reverse genetics systems have been established for only a few plant NSR viruses, and no infectious reverse genetics system is yet widely available for plant dsRNA reoviruses in either plant hosts or insect vectors. This limitation restricts the use of gene-deletion or site-directed viral mutants to rigorously define the functions of viral genes during infection and transmission. The recent development of reverse genetics systems for plant rhabdoviruses, including BYSMV, northern cereal mosaic virus (NCMV), RSMV, and MMV [9,64–67], has greatly advanced studies of tripartite interactions among viruses, host plants, and insect vectors. GFP- and/or RFP-expressing BYSMV, NCMV, RSMV, and MMV clones enable real-time and unbiased monitoring of viral infection dynamics in both plants and insect vectors [9,65,66,68,69]. More importantly, individual viral genes can be mutated to uncover their authentic functions in genetically defined systems [68,70,71]. Reverse genetics systems have also been established for some plant bunyaviruses, such as TSWV, providing valuable tools for studying virus–plant–vector interactions [72]. For RSV, infectious clones remain unavailable, although minireplicon systems have been developed [73,74]. Reverse genetics systems remain unavailable for plant dsRNA reoviruses such as RDV, RBSDV, and SRBSDV. However, the successful rescue of several animal dsRNA viruses provides valuable conceptual and technical templates for developing comparable reverse genetics platforms for plant dsRNA viruses [75]. These advances in reverse genetics will present new opportunities for extensive investigations of virus–insect interactions.

## Efficient CRISPR-based systems are needed for the genetic manipulation of insect vectors

Most nonmodel insect vectors still lack reliable genetic transformation systems, which limits transgenic overexpression and gene knockout approaches for functional validation of vector genes. As a result, dsRNA-mediated RNA interference is widely used to silence vector genes, whereas CRISPR-based knockouts, although achieved in planthoppers and aphids, remain labor-intensive, technically demanding, and often inefficient [76–78]. A major challenge is that many insect vectors have small bodies and fragile embryos, making transformation methods established in model insects such as *Drosophila* difficult to apply directly.

Virus-mediated expression and genome editing systems may provide a powerful alternative strategy for functional studies in insect vectors. Plant rhabdovirus-based vectors, including BYSMV and RSMV, have been developed to express foreign proteins in several planthopper and leafhopper vectors, including *Nilaparvata lugens*, *Laodelphax striatellus*, *Sogatella furcifera*, and *Recilia dorsalis* [65,79]. The BYSMV vector has been developed into a delivery system for Virus-induced Genome Editing in Tiller (ViGET) for heritable and transgene-free genome editing in monocot crops [80]. Extending similar virus-mediated genome editing strategies to insect vectors would be highly valuable, as it could substantially lower the technical barrier for functional genetic studies of vector genes involved in virus acquisition, circulation, replication, and transmission, as well as for controlling insect-borne viral diseases. For example, knockout of the aphid acrostyle receptor Stylin-01, required for CaMV P2 binding, severely impaired CaMV transmission, demonstrating that vector genome editing can disrupt viral receptor sites and limit virus spread [81].

## From single virus–vector systems to multidimensional interaction networks

Current understanding of virus-mediated manipulation of insect vector behavior is still largely derived from laboratory studies using single virus–plant–vector systems. However, under field conditions, host plants and insect vectors are frequently challenged by mixed infections involving multiple plant viruses, insect-specific viruses, and microbial symbionts. For example, several rice-infecting viruses share both host plants and insect vectors. RDV and RYSV are transmitted by the green rice leafhopper *Nephotettix cincticeps* [51,35], whereas rice gall dwarf virus (RGDV) and RSMV are transmitted by the leafhopper *Recilia dorsalis* [82]. Such overlap in host and vector ranges raises an important question: do co-occurring plant viruses cooperate, compete, or independently manipulate the same plant–vector interface under mixed-infection conditions?

Beyond plant virus–plant virus interactions, insect-associated symbiotic viruses can also modulate plant virus transmission. The leafhopper symbiotic virus *Recilia dorsalis* bunyavirus (RdBV) enhances RSMV transmission by suppressing the insect E3 ubiquitin ligase RdSina-mediated degradation of the RSMV phosphoprotein, thereby promoting RSMV accumulation [83]. Similarly, the leafhopper symbiotic virus *Recilia* *dorsalis* filamentous virus (RdFV) promotes paternal vertical transmission of RGDV, with its capsid protein interacting with RGDV P8 to facilitate viral movement through the male reproductive pathway [84]. Bacterial symbionts add another layer of complexity to plant virus–vector interactions. RDV exploits the obligate symbiont *Sulcia* in green rice leafhoppers, with its outer capsid protein P2 directly interacting with a *Sulcia* outer membrane protein to access the symbiont-associated oocyte entry route for transovarial transmission [85]. Conversely, *Wolbachia* suppresses rice ragged stunt virus (RRSV) infection and transmission in *Nilaparvata lugens* [86], indicating that bacterial symbionts can either facilitate or restrict plant virus transmission. Together, these findings suggest that virus-mediated vector manipulation is not a simple one-virus–one-vector process, but rather a multidimensional network shaped by co-infecting plant viruses, insect-associated viruses, bacterial symbionts, and host plant responses.

## Concluding remarks

Insect vector transmission is essential for the infection cycles of most plant viruses. Accumulating evidence has revealed that plant viruses are not passive passengers but actively modulate the feeding behavior, physiology, and development of insect vectors to promote efficient transmission through both indirect plant-mediated and direct vector-targeted mechanisms. Deciphering the molecular basis of these interactions will not only advance our understanding of virus–plant–insect coevolution but also facilitate the development of novel strategies to control insect vectors and interrupt viral spread. Future studies should establish robust viral reverse genetics systems and efficient insect genetic manipulation tools, while extending research from simplified laboratory models to field-relevant ecological contexts involving mixed infections, vector microbiota, environmental variation, and multitrophic interactions.

## References

[1] Gao D, Jiang R, Wang Z, Niu J, Wang G, Wang Y, et al. DENV2 and ZIKV modulate the feeding behavior of *Aedes aegypti* by altering the tyrosine-dopamine pathway. mBio. 2025;16(6):e0396824. doi: 10.1128/mbio.03968-24 40298398 PMC12153320

[2] Hogenhout SA, Ammar E-D, Whitfield AE, Redinbaugh MG. Insect vector interactions with persistently transmitted viruses. Annu Rev Phytopathol. 2008;46:327–59. doi: 10.1146/annurev.phyto.022508.092135 18680428

[3] Lefeuvre P, Martin DP, Elena SF, Shepherd DN, Roumagnac P, Varsani A. Evolution and ecology of plant viruses. Nat Rev Microbiol. 2019;17(10):632–44. doi: 10.1038/s41579-019-0232-3 31312033

[4] Blanc S, Drucker M, Uzest M. Localizing viruses in their insect vectors. Annu Rev Phytopathol. 2014;52:403–25. doi: 10.1146/annurev-phyto-102313-045920 24996011

[5] NG JCK, PERRY KL. Transmission of plant viruses by aphid vectors. Mol Plant Pathol. 2004;5(5):505–11. doi: 10.1111/j.1364-3703.2004.00240.x 20565624 10.1111/j.1364-3703.2004.00240.x

[6] Sarwar M. Insects as transport devices of plant viruses. App Plant Virol. 2020:381–402. doi: 10.1016/b978-0-12-818654-1.00027-x

[7] Whitfield AE, Rotenberg D. Disruption of insect transmission of plant viruses. Curr Opin Insect Sci. 2015;8:79–87. doi: 10.1016/j.cois.2015.01.009 32846687

[8] Cao Q, Xu W-Y, Gao Q, Jiang Z-H, Liu S-Y, Fang X-D, et al. Transmission characteristics of barley yellow striate mosaic virus in its planthopper vector *Laodelphax striatellus*. Front Microbiol. 2018;9:1419. doi: 10.3389/fmicb.2018.01419 30008708 PMC6034074

[9] Gao Q, Xu W-Y, Yan T, Fang X-D, Cao Q, Zhang Z-J, et al. Rescue of a plant cytorhabdovirus as versatile expression platforms for planthopper and cereal genomic studies. New Phytol. 2019;223(4):2120–33. doi: 10.1111/nph.15889 31059138

[10] Zhao P, Sun X, Li P, Sun J, Yue Y, Wei J, et al. Infection characteristics of rice stripe mosaic virus in the body of the vector leafhoppers. Front Microbiol. 2019;9:3258. doi: 10.3389/fmicb.2018.03258 30671049 PMC6331539

[11] Deshoux M, Monsion B, Uzest M. Insect cuticular proteins and their role in transmission of phytoviruses. Curr Opin Virol. 2018;33:137–43. doi: 10.1016/j.coviro.2018.07.015 30245214 PMC6291435

[12] Li R, Weldegergis BT, Li J, Jung C, Qu J, Sun Y, et al. Virulence factors of geminivirus interact with MYC2 to subvert plant resistance and promote vector performance. Plant Cell. 2014;26(12):4991–5008. doi: 10.1105/tpc.114.133181 25490915 PMC4311212

[13] Zhao P, Yao X, Cai C, Li R, Du J, Sun Y, et al. Viruses mobilize plant immunity to deter nonvector insect herbivores. Sci Adv. 2019;5(8):eaav9801. doi: 10.1126/sciadv.aav9801 31457079 PMC6703867

[14] Shi X-B, Yue H, Wei Y, Preisser EL, Wang P, Du J, et al. Neophytadiene, a plant specialized metabolite, mediates the virus-vector-plant tripartite interactions. Adv Sci. 2025;12(22):e2416891. doi: 10.1002/advs.202416891 40178133 PMC12165043

[15] Yu X, Zhu Y, Yin G, Wang Y, Shi X, Cheng G. Exploiting hosts and vectors: viral strategies for facilitating transmission. EMBO Rep. 2024;25(8):3187–201. doi: 10.1038/s44319-024-00214-6 39048750 PMC11315993

[16] Pan L-L, Miao H, Wang Q, Walling LL, Liu S-S. Virus-induced phytohormone dynamics and their effects on plant-insect interactions. New Phytol. 2021;230(4):1305–20. doi: 10.1111/nph.17261 33555072 PMC8251853

[17] Eigenbrode SD, Bosque-Pérez NA, Davis TS. Insect-borne plant pathogens and their vectors: ecology, evolution, and complex interactions. Annu Rev Entomol. 2018;63:169–91. doi: 10.1146/annurev-ento-020117-043119 28968147

[18] Bragard C, Caciagli P, Lemaire O, Lopez-Moya JJ, MacFarlane S, Peters D, et al. Status and prospects of plant virus control through interference with vector transmission. Annu Rev Phytopathol. 2013;51:177–201. doi: 10.1146/annurev-phyto-082712-102346 23663003

[19] Blanc S, Michalakis Y. Manipulation of hosts and vectors by plant viruses and impact of the environment. Curr Opin Insect Sci. 2016;16:36–43. doi: 10.1016/j.cois.2016.05.007 27720048

[20] Mauck KE. Variation in virus effects on host plant phenotypes and insect vector behavior: what can it teach us about virus evolution? Curr Opin Virol. 2016;21:114–23. doi: 10.1016/j.coviro.2016.09.002 27644035

[21] Ye J, Zhang L, Zhang X, Wu X, Fang R. Plant defense networks against insect-borne pathogens. Trends Plant Sci. 2021;26(3):272–87. doi: 10.1016/j.tplants.2020.10.009 33277186

[22] Whitfield AE, Falk BW, Rotenberg D. Insect vector-mediated transmission of plant viruses. Virology. 2015;479–480:278–89. doi: 10.1016/j.virol.2015.03.026 25824478

[23] He Y-Z, Wang Y-M, Yin T-Y, Fiallo-Olivé E, Liu Y-Q, Hanley-Bowdoin L, et al. A plant DNA virus replicates in the salivary glands of its insect vector via recruitment of host DNA synthesis machinery. Proc Natl Acad Sci U S A. 2020;117(29):16928–37. doi: 10.1073/pnas.1820132117 32636269 PMC7382290

[24] Sánchez-Campos S, Rodríguez-Negrete EA, Cruzado L, Grande-Pérez A, Bejarano ER, Navas-Castillo J, et al. Tomato yellow leaf curl virus: no evidence for replication in the insect vector *Bemisia tabaci*. Sci Rep. 2016;6:30942. doi: 10.1038/srep30942 27476582 PMC4967916

[25] Hogenhout SA, Ammar E-D, Whitfield AE, Redinbaugh MG. Insect vector interactions with persistently transmitted viruses. Annu Rev Phytopathol. 2008;46:327–59. doi: 10.1146/annurev.phyto.022508.092135 18680428

[26] Yu J, Zhao W, Chen X, Lu H, Xiao Y, Li Q, et al. A plant virus manipulates the long-winged morph of insect vectors. Proc Natl Acad Sci U S A. 2024;121(3):e2315341121. doi: 10.1073/pnas.2315341121 38190519 PMC10801844

[27] Ai S, Luo C, Yao X, Qu W, Wang Y, Zhang T, et al. Manipulation of host-plant preference by virus-induced changes to its insect vector’s olfactory system. Curr Biol. 2025;35(15):3587-3600.e6. doi: 10.1016/j.cub.2025.05.064 40920647

[28] Gao D-M, Qiao J-H, Gao Q, Zhang J, Zang Y, Xie L, et al. A plant cytorhabdovirus modulates locomotor activity of insect vectors to enhance virus transmission. Nat Commun. 2023;14(1):5754. doi: 10.1038/s41467-023-41503-3 37717061 PMC10505171

[29] Walker PJ, Freitas-Astúa J, Bejerman N, Blasdell KR, Breyta R, Dietzgen RG, et al. ICTV virus taxonomy profile: rhabdoviridae 2022. J Gen Virol. 2022;103(6):001689. doi: 10.1099/jgv.0.001689 35723908 PMC12662027

[30] Rubino L, Abrahamian P, An W, Aranda MA, Ascencio-Ibañez JT, Bejerman N, et al. Summary of taxonomy changes ratified by the International Committee on Taxonomy of Viruses from the Plant Viruses Subcommittee, 2025. J Gen Virol. 2025;106(7). doi: 10.1099/jgv.0.002114 40711908 10.1099/jgv.0.002114 PMC12451643

[31] Debat H, Sidharthan VK, Viswanathan R, Ramos-González PL, Freitas-Astúa J, Bhat AI, et al. Taxonomy of family: Rhabdoviridae. In: Taxonomy and classification of plant viruses and viroids. Springer Nature Singapore; 2026. p. 885–945. doi: 10.1007/978-981-97-8408-0_30

[32] Ammar E-D, Tsai C-W, Whitfield AE, Redinbaugh MG, Hogenhout SA. Cellular and molecular aspects of rhabdovirus interactions with insect and plant hosts. Annu Rev Entomol. 2009;54:447–68. doi: 10.1146/annurev.ento.54.110807.090454 18793103

[33] Todd JC, Ammar E-D, Redinbaugh MG, Hoy C, Hogenhout SA. Plant host range and leafhopper transmission of maize fine streak virus. Phytopathology. 2010;100(11):1138–45. doi: 10.1094/PHYTO-05-10-0144 20649417

[34] Ammar E-D, Hogenhout SA. A neurotropic route for maize mosaic virus (Rhabdoviridae) in its planthopper vector *Peregrinus maidis*. Virus Res. 2008;131(1):77–85. doi: 10.1016/j.virusres.2007.08.010 17928084

[35] Wang H, Wang J, Zhang Q, Zeng T, Zheng Y, Chen H, et al. Rice yellow stunt nucleorhabdovirus matrix protein mediates viral axonal transport in the central nervous system of its insect vector. Front Microbiol. 2019;10:939. doi: 10.3389/fmicb.2019.00939 31143161 PMC6521124

[36] Fooks AR, Cliquet F, Finke S, Freuling C, Hemachudha T, Mani RS. Rabies. Nat Rev Dis Primers. 2017;3(1):17091. doi: 10.1038/nrdp.2017.91 29188797 10.1038/nrdp.2017.91

[37] Wang H, Liu Y, Mo L, Huo C, Wang Z, Zhong P, et al. A neuron-specific antiviral mechanism modulates the persistent infection of rice rhabdoviruses in leafhopper vectors. Front Microbiol. 2020;11:513. doi: 10.3389/fmicb.2020.00513 32362876 PMC7180231

[38] Drolet BS, Campbell CL, Stuart MA, Wilson WC. Vector competence of *Culicoides sonorensis* (Diptera: Ceratopogonidae) for vesicular stomatitis virus. J Med Entomol. 2005;42(3):409–18. doi: 10.1603/0022-2585(2005)042[0409:vcocsd]2.0.co;2 15962795

[39] Yan T, Zhu J-R, Di D, Gao Q, Zhang Y, Zhang A, et al. Characterization of the complete genome of Barley yellow striate mosaic virus reveals a nested gene encoding a small hydrophobic protein. Virology. 2015;478:112–22. doi: 10.1016/j.virol.2014.12.042 25666524

[40] Gao D-M, Zhang Z-J, Qiao J-H, Gao Q, Zang Y, Xu W-Y, et al. A rhabdovirus accessory protein inhibits jasmonic acid signaling in plants to attract insect vectors. Plant Physiol. 2022;190(2):1349–64. doi: 10.1093/plphys/kiac319 35771641 PMC9516739

[41] Chen S, Zhong X, Wang Z, Chen B, Huang X, Xu S, et al. Rice stripe mosaic virus hijacks rice heading-related gene to promote the overwintering of its insect vector. J Integr Plant Biol. 2024;66(9):2000–16. doi: 10.1111/jipb.13722 38923382

[42] Kormelink R, Verchot J, Tao X, Desbiez C. The bunyavirales: the plant-infecting counterparts. Viruses. 2021;13(5):842. doi: 10.3390/v13050842 34066457 PMC8148189

[43] Parrish WB. The origin, morphology, and innervation of aphid stylets (Homoptera). Ann Entomol Soc. 1967;60(1):273–6. doi: 10.1093/aesa/60.1.273 38923382

[44] Yang Z, Wu W, Xu Z, Li Y, Zhang H, Li L, et al. Arbovirus suppression of a lectin protein-mediated broad-spectrum resistance enhances herbivorous vector performance and viral transmission. Nat Commun. 2025;16(1):6873. doi: 10.1038/s41467-025-62233-8 40715108 PMC12297515

[45] Xu H-J, Xue J, Lu B, Zhang X-C, Zhuo J-C, He S-F, et al. Two insulin receptors determine alternative wing morphs in planthoppers. Nature. 2015;519(7544):464–7. doi: 10.1038/nature14286 25799997

[46] Montasser MS, Tousignant ME, Kaper JM. Viral Satellite RNAs for the prevention of cucumber mosaic virus (CMV) disease in field-grown pepper and melon plants. Plant Dis. 1998;82(12):1298–303. doi: 10.1094/PDIS.1998.82.12.1298 30845460

[47] Shimura H, Pantaleo V, Ishihara T, Myojo N, Inaba J, Sueda K, et al. A viral satellite RNA induces yellow symptoms on tobacco by targeting a gene involved in chlorophyll biosynthesis using the RNA silencing machinery. PLoS Pathog. 2011;7(5):e1002021. doi: 10.1371/journal.ppat.1002021 21573143 PMC3088725

[48] Jayasinghe WH, Kim H, Nakada Y, Masuta C. A plant virus satellite RNA directly accelerates wing formation in its insect vector for spread. Nat Commun. 2021;12(1):7087. doi: 10.1038/s41467-021-27330-4 34873158 PMC8648847

[49] Liu Q, Wang Q, Li Q, Wang W, Li Q, Peng Z, et al. Arboviruses manipulate rice’s volatile emissions, protecting insect vectors from natural enemies in the field. Sci Adv. 2026;12(2):eaeb5215. doi: 10.1126/sciadv.aeb5215 41499511 PMC12778065

[50] Wu X, Xu S, Zhao P, Zhang X, Yao X, Sun Y, et al. The Orthotospovirus nonstructural protein NSs suppresses plant MYC-regulated jasmonate signaling leading to enhanced vector attraction and performance. PLoS Pathog. 2019;15(6):e1007897. doi: 10.1371/journal.ppat.1007897 31206553 PMC6598649

[51] Wei T, Li Y. Rice reoviruses in insect vectors. Annu Rev Phytopathol. 2016;54:99–120. doi: 10.1146/annurev-phyto-080615-095900 27296147

[52] Chang X, Wang F, Fang Q, Chen F, Yao H, Gatehouse AMR, et al. Virus-induced plant volatiles mediate the olfactory behaviour of its insect vectors. Plant Cell Environ. 2021;44(8):2700–15. doi: 10.1111/pce.14069 33866575

[53] Wang H, Xu D, Pu L, Zhou G. Southern rice black-streaked dwarf virus alters insect vectors’ host orientation preferences to enhance spread and increase rice ragged stunt virus co-infection. Phytopathology. 2014;104(2):196–201. doi: 10.1094/PHYTO-08-13-0227-R 24047253

[54] Lu G, Zhang T, He Y, Zhou G. Virus altered rice attractiveness to planthoppers is mediated by volatiles and related to virus titre and expression of defence and volatile-biosynthesis genes. Sci Rep. 2016;6:38581. doi: 10.1038/srep38581 27924841 PMC5141440

[55] Zhao Y, Cao X, Zhong W, Zhou S, Li Z, An H, et al. A viral protein orchestrates rice ethylene signaling to coordinate viral infection and insect vector-mediated transmission. Mol Plant. 2022;15(4):689–705. doi: 10.1016/j.molp.2022.01.006 35032687

[56] Mori K, Sakano H. Olfactory circuitry and behavioral decisions. Annu Rev Physiol. 2021;83:231–56. doi: 10.1146/annurev-physiol-031820-092824 33228453

[57] Wang H, Ye J. Vector biology: Virus hijacks vector olfaction to target new hosts. Curr Biol. 2025;35(15):R752–5. doi: 10.1016/j.cub.2025.06.035 40763698

[58] Wang X-W, Blanc S. Insect transmission of plant single-stranded DNA viruses. Annu Rev Entomol. 2021;66:389–405. doi: 10.1146/annurev-ento-060920-094531 32931313

[59] Ryckebusch F, Peterschmitt M, Granier M, Sauvion N. Alfalfa leaf curl virus is efficiently acquired by its aphid vector *Aphis craccivora* but inefficiently transmitted. J Gen Virol. 2021;102(2):001516. doi: 10.1099/jgv.0.001516 33210990 PMC8116941

[60] Nie Z, Guo Z, Qin X, Liu Q, Zhou M, Ye J, et al. An efficient beet severe curly top virus-based vector for VIGS and HIGS in *Beta vulgaris*. Plant Biotechnol J. 2026;24(2):731–3. doi: 10.1111/pbi.70345 40986430 PMC12906799

[61] Fang Y, Jiao X, Xie W, Wang S, Wu Q, Shi X, et al. Tomato yellow leaf curl virus alters the host preferences of its vector *Bemisia tabaci*. Sci Rep. 2013;3:2876. doi: 10.1038/srep02876 24096821 PMC3791452

[62] Liang P, Zeng Y, Ning J, Wu X, Wang W, Ren J, et al. A plant virus manipulates both its host plant and the insect that facilitates its transmission. Sci Adv. 2025;11(9):eadr4563. doi: 10.1126/sciadv.adr4563 40020061 PMC11870061

[63] Wang S, Guo H, Ge F, Sun Y. Apoptotic neurodegeneration in whitefly promotes the spread of TYLCV. eLife. 2020;9:e56168. doi: 10.7554/eLife.56168 32729829 PMC7392610

[64] Fang X-D, Qiao J-H, Zang Y, Gao Q, Xu W-Y, Gao D-M, et al. Developing reverse genetics systems of northern cereal mosaic virus to reveal superinfection exclusion of two cytorhabdoviruses in barley plants. Mol Plant Pathol. 2022;23(5):749–56. doi: 10.1111/mpp.13188 35124878 PMC8995060

[65] Wu K, Chen B, Liu Q, Sun K, Shao Y, Mijiti M, et al. Development of a plant rhabdovirus-based versatile vector for gene function studies in leafhoppers and rice. J Exp Bot. 2026;:erag082. doi: 10.1093/jxb/erag082 41685868

[66] Kanakala S, Xavier CAD, Martin KM, Tran HH, Redinbaugh MG, Whitfield AE. Rescue of the first alphanucleorhabdovirus entirely from cloned complementary DNA: an efficient vector for systemic expression of foreign genes in maize and insect vectors. Mol Plant Pathol. 2023;24(7):788–800. doi: 10.1111/mpp.13273 36239302 PMC10257042

[67] Qiao J-H, Gao Q, Wang X-B. Virus-induced genome editing: toward crop breeding applications. Trends Plant Sci. 2026;:S1360-1385(26)00022-1. doi: 10.1016/j.tplants.2026.01.007 41813543

[68] Zang Y, Qiao J-H, Liu D-S, Gao D-M, Zhang X-W, Pan T-T, et al. The barley m6A demethylase HvALKBH1B undergoes phase separation to enhance immunity to a plant rhabdovirus. Plant Cell. 2025;37(9):koaf210. doi: 10.1093/plcell/koaf210 40857584

[69] Zang Y, Fang XD, Qiao JH, Gao Q, Wang XB. Reverse genetics systems of plant negative-strand RNA viruses are difficult to be developed but powerful for virus-host interaction studies and virus-based vector applications. Phytopathol Res. 2020;2(1):29. doi: 10.1186/s42483-020-00068-5

[70] Ding Z-H, Gao Q, Tong X, Xu W-Y, Ma L, Zhang Z-J, et al. MAPKs trigger antiviral immunity by directly phosphorylating a rhabdovirus nucleoprotein in plants and insect vectors. Plant Cell. 2022;34(8):3110–27. doi: 10.1093/plcell/koac143 35567529 PMC9338794

[71] Gao Q, Zang Y, Qiao J-H, Zhang Z-Y, Wang Y, Han C-G, et al. The plant rhabdovirus viroporin P9 facilitates insect-mediated virus transmission in barley. Plant Cell. 2024;36(9):3483–97. doi: 10.1093/plcell/koae162 38819305 PMC11371171

[72] Feng M, Cheng R, Chen M, Guo R, Li L, Feng Z, et al. Rescue of tomato spotted wilt virus entirely from complementary DNA clones. Proc Natl Acad Sci U S A. 2020;117(2):1181–90. doi: 10.1073/pnas.1910787117 31879355 PMC6969498

[73] Feng M, Li L, Cheng R, Yuan Y, Dong Y, Chen M, et al. Development of a mini-replicon-based reverse-genetics system for rice stripe tenuivirus. J Virol. 2021;95(14):e0058921. doi: 10.1128/JVI.00589-21 33952642 PMC8223943

[74] Zhang X, Sun K, Liang Y, Wang S, Wu K, Li Z. Development of rice stripe tenuivirus minireplicon reverse genetics systems suitable for analyses of viral replication and intercellular movement. Front Microbiol. 2021;12:655256. doi: 10.3389/fmicb.2021.655256 33833749 PMC8021733

[75] Kobayashi T, Antar AAR, Boehme KW, Danthi P, Eby EA, Guglielmi KM, et al. A plasmid-based reverse genetics system for animal double-stranded RNA viruses. Cell Host Microbe. 2007;1(2):147–57. doi: 10.1016/j.chom.2007.03.003 18005692 PMC2034303

[76] Le Trionnaire G, Tanguy S, Hudaverdian S, Gleonnec F, Richard G, Cayrol B, et al. An integrated protocol for targeted mutagenesis with CRISPR-Cas9 system in the pea aphid. Insect Biochem Mol Biol. 2019;110:34–44. doi: 10.1016/j.ibmb.2019.04.016 31015023

[77] Zhang M-Q, Gong L-L, Zhao Y-Q, Ma Y-F, Long G-J, Guo H, et al. Efficient DIPA-CRISPR-mediated knockout of an eye pigment gene in the white-backed planthopper, *Sogatella furcifera*. Insect Sci. 2024;31(4):1015–25. doi: 10.1111/1744-7917.13286 37919237

[78] Xue W-H, Xu N, Yuan X-B, Chen H-H, Zhang J-L, Fu S-J, et al. CRISPR/Cas9-mediated knockout of two eye pigmentation genes in the brown planthopper, *Nilaparvata lugens* (Hemiptera: Delphacidae). Insect Biochem Mol Biol. 2018;93:19–26. doi: 10.1016/j.ibmb.2017.12.003 29241845

[79] Xu W-Y, Fang X-D, Cao Q, Gao Q, Gao D-M, Qiao J-H, et al. A cytorhabdovirus-based expression vector in *Nilaparvata lugens*, *Laodelphax striatellus*, and *Sogatella furcifera*. Insect Biochem Mol Biol. 2022;140:103703. doi: 10.1016/j.ibmb.2021.103703 34933088

[80] Qiao J-H, Zang Y, Gao Q, Liu S, Zhang X-W, Hu W, et al. Transgene- and tissue culture-free heritable genome editing using RNA virus-based delivery in wheat. Nat Plants. 2025;11(7):1252–9. doi: 10.1038/s41477-025-02023-8 40562814

[81] Fu Y, Deshoux M, Cayrol B, Le Blaye S, Achard E, Hudaverdian S, et al. Stylet cuticular gene-directed mutagenesis impairs the pea aphid vector capacity to transmit a plant virus. PLoS Pathog. 2025;21(5):e1013192. doi: 10.1371/journal.ppat.1013192 40408450 PMC12140417

[82] Jia D, Luo G, Shi W, Liu Y, Liu H, Zhang X, et al. Rice gall dwarf virus promotes the propagation and transmission of rice stripe mosaic virus by co-infected insect vectors. Front Microbiol. 2022;13:834712. doi: 10.3389/fmicb.2022.834712 35222343 PMC8874222

[83] Wang H, Zhang J, Liu R, Li Y, Du Y, Wei T. An insect symbiotic virus promotes the transmission of a phytoarbovirus via inhibiting E3 ubiquitin ligase Sina. PLoS Pathog. 2025;21(5):e1013178. doi: 10.1371/journal.ppat.1013178 40440302 PMC12121772

[84] Wan J, Liang Q, Zhang R, Cheng Y, Wang X, Wang H, et al. Arboviruses and symbiotic viruses cooperatively hijack insect sperm-specific proteins for paternal transmission. Nat Commun. 2023;14(1):1289. doi: 10.1038/s41467-023-36993-0 36894574 PMC9998617

[85] Jia D, Mao Q, Chen Y, Liu Y, Chen Q, Wu W, et al. Insect symbiotic bacteria harbour viral pathogens for transovarial transmission. Nat Microbiol. 2017;2:17025. doi: 10.1038/nmicrobiol.2017.25 28263320

[86] Gong J-T, Li Y, Li T-P, Liang Y, Hu L, Zhang D, et al. Stable introduction of plant-virus-inhibiting *Wolbachia* into planthoppers for rice protection. Curr Biol. 2020;30(24):4837-4845.e5. doi: 10.1016/j.cub.2020.09.033 33035486

